# Female Tendons are from Venus and Male Tendons are from Mars, But Does it Matter for Tendon Health?

**DOI:** 10.1007/s40279-024-02056-7

**Published:** 2024-07-29

**Authors:** Gerard McMahon, Jill Cook

**Affiliations:** 1 Sport and Exercise Sciences Research Institute, School of Sport, Ulster University, York Street, Belfast, North Ireland BT15 1ED UK; 2 La Trobe Sport and Exercise Medicine Research Centre, La Trobe University, Melbourne, VIC Australia

## Abstract

Tendons play fundamental roles in the execution of human movement and therefore understanding tendon function, health and disease is important for everyday living and sports performance. The acute mechanical behavioural and physiological responses to short-term loading of tendons, as well as more chronic morphological and mechanical adaptations to longer term loading, differ between sexes. This has led some researchers to speculate that there may be a sex-specific injury risk in tendons. However, the link between anatomical, physiological and biomechanical sex-specific differences in tendons and their contributory role in the development of tendon disease injuries has not been critically evaluated. This review outlines the evidence surrounding the sex-specific physiological and biomechanical responses and adaptations to loading and discusses how this evidence compares to clinical evidence on tendon injuries and rehabilitation in the Achilles and patellar tendons in humans. Using the evidence available in both sports science and medicine, this may provide a more holistic understanding to improve our ability to enhance human tendon health and performance in both sexes.

## Key Points

**Table:** 

There are several key differences in tendon mechanical properties and morphology, physiological responses to loading and tendon adaptation between sexes.
Current limitations in study designs and methods of key variable assessments present in the literature restrict the ability to draw strong conclusions between injury risk differences based on sex-specific tendon differences.
Based on current information, links between sex-specific tendon differences and the link to injury risk differences between sexes are unwarranted or at best, premature. Future research should address the limitations of current study designs and measures.

## Introduction

There are clear sex-based differences in tendon morphology, physiology and mechanical properties [1]. Female individuals display a lower level of tendon adaptability to loading than male individuals, as well as lower tendon stiffness and a smaller cross-sectional area (CSA) [1]. Physiologically, the molecular responsiveness of tendon tissues to loading also appears to differ between male and female individuals [2, 3]. These are summarised in Table 1.

**Table 1:** Summary of sex-specific differences in tendon properties, responses and adaptation linked to potentially divergent effects on tendon health

Tendon	Tendon dimensions, tissue content and mechanical properties	Acute physiological and mechanical behavioural responses to exercise loading	Differences in adaptation following exercise training or habitual activity
Achilles (free tendon) or gastrocnemius-tendon aponeurosis	AT CSA [14] AT length [14] Force, stress, strain, stiffness, Young’s modulus and hysteresis [5, 9, 11, 12]	AT stiffness, Young’s modulus and hysteresis [5, 11, 12] AT elongation [5]	AT CSA [4]
Patellar	PT CSA [19–23] PT length [20–23] Maximal stress, maximal stiffness, Young’s modulus Stiffness at standardised force level [19] Absolute collagen dry mass [35] Collagen per wet weight [35] Type III collagen mRNA [3] MMP-3 mRNA [3]	Resting collagen FSR [2] Post-exercise recovery period (24–72 h) collagen FSR [34]	PT CSA [4] Δ↑ PT stiffness at either end of the tendon force–elongation curve [20, 46] PT stiffness (older only, [20, 22])

*AT* Achilles tendon, *CSA* cross-sectional area, *FSR* fractional synthetic rate, *MMP-3* metalloproteinase-3, *mRNA* messenger RNA, *PT* patellar tendon

The sex differences in mechanical properties, morphology, strain magnitude, synthesis of tendon tissue and tendon structural health may impact tendon injury risk in male individuals compared with female individuals [1, 4, 5]. Patellar tendinopathy (and tendon pathology), a condition of younger athletes, is twice as prevalent in male individuals than female individuals [6, 7]. The prevalences of Achilles tendon (AT) pathology and pain are no different between male and female individuals [8].

This Current Opinion article focuses on the AT and patellar tendon (PT) and examines the evidence for sex differences in tendon physiology, biomechanics, and the response to load between human male and female individuals. The article evaluates the clinical implications of these differences. Computerised electronic databases PubMed/MEDLINE, SPORTDiscus and Google Scholar were searched from March to June 2023 to identify relevant literature using the following keywords; tendon, human, sex, male, female, patella, Achilles, tendinopathy, mechanical properties, material properties, strain, stiffness, stress, Young’s Modulus, cross-sectional area, adaptation, ultrasound, MRI.

## What are the Tendon Morphological and Mechanical Differences Between Men and Women?

### Morphological Properties of Tendons in Men and Women

Although on average male individuals have greater absolute body mass and are taller than female individuals, and greater absolute muscle mass will influence tendon mass, these factors are not accounted for when describing the differences in mechanical properties of tendon between male and female individuals [9–12]. Sex may also affect tendon properties through sex hormones, menarche, menopause and oral contraceptive use [13].

Morphological differences between male and female individuals may also impact on the properties of tendons. Male individuals have a significantly greater AT CSA and length than female individuals [14]. Skeletal morphology (pelvic width) has also been associated with some tendinopathies of the hip. Gluteal tendinopathy is five to six times more prevalent in female individuals than male individuals. This can be explained by pelvis and femur structure, as female individuals with a wider pelvis place a greater compressive load on the gluteal tendons than male individuals [15].

Male and female individuals also reach puberty at different ages and mature at different rates during puberty. As tendons likely accumulate their mass before and during puberty, similar to bone, this may have an effect on morphological properties. It is likely that female individuals have a shorter stimulus time to form tendon mass than male individuals as they reach puberty earlier, which may also be related to their lower muscle and body mass. There is little evidence that tendon mass increases with loading after puberty [16], although pathology in a tendon does increase the mass of normal tendon tissue in an unknown way [17].

### Mechanical and Material Properties of Tendons in Men and Women

The medial gastrocnemius tendon-aponeurosis elongation values in vivo were significantly greater in female individuals compared with male individuals [9]. However, maximum strain, stiffness, Young’s modulus and hysteresis were all significantly lower in female individuals than male individuals [9]. Similar findings in the morphology and mechanical properties are seen in the free AT or gastrocnemius tendon. Male individuals have greater AT force, stress, strain, hysteresis, stiffness and modulus than young age-matched female individuals [5, 11, 12]. However, it should be noted that the sex differences in AT mechanical and material properties have not been directly assessed in older populations, although some evidence suggests that a negative relationship between mechanical properties in the AT and age exists in female individuals but not in male individuals [18].

The in vivo mechanical properties of the PT were measured in ten young male individuals and ten young female individuals [19]. The PT length was similar between sexes, but significantly thinner in female individuals than in male individuals [19]. Although maximal PT displacement and maximal total PT strain were similar between the groups, male individuals had significantly greater maximal total stress than female individuals [19]. As a result, mean PT stiffness, maximal stiffness and Young’s modulus were significantly greater in male individuals compared with female individuals. To account for the large differences in strength (i.e. maximal voluntary contraction), PT mechanical properties were calculated at a standardised force level. Patellar tendon stiffness was significantly greater (57%) and stress significantly lower (− 22%) in male individuals compared with female individuals [19]. Significantly greater PT CSAs, lengths, stiffness and modulus are reported in both younger and older male individuals compared with younger and older female individuals at baseline [20–23]. Another study demonstrated that in a single isolated human PT fascicle, young male individuals had significantly greater peak stress and tangent modulus than female individuals [1]. The evidence for both PT and AT supports the absolute mechanical and material properties of female individuals’ tendons being significantly lower than men’s. However, work by O’Brien et al. [24] suggests that when PT length is normalised to fascicle length of any of the four quadriceps muscles, there are no differences between male and female individuals. Therefore, more research is needed to determine if a true sex difference exists with regard to tendon mechanical properties and morphology.

Joseph et al. [5] and Lepley et al. [12] speculated that the increased compliance of female tendons at baseline and following exercise may render female individuals less susceptible to tendon injury than male individuals as male individuals may generate higher muscle and tendon forces, leading to higher exposure of tissue overload [12]. However, these authors did not link these observations to any specific pathophysiological model of the development of tendinopathy or the risk of rupture. Higher muscle and tendon forces in male individuals are not necessarily an issue, as high muscle and tendon forces can be generated but not necessarily result in high overall tendon strain [25]. As previously highlighted, it is indeed female individuals who ultimately display higher strains. Indeed, other research from Arampatzis et al. [26] suggests that routinely exercising closer to ultimate strain reduces the safety factor (ratio of operating strain to ultimate strain), possibly increasing the injury risk. This is mainly based on increased microtrauma or injury to tendons observed at high strains (≥ 9%) in primarily animal data [27] or ex vivo mechanical testing of human AT [28]. This would therefore imply that based on baseline mechanical properties and mechanical responses to loading, it would be female individuals who would be at an increased injury risk. Mersmann et al. [29] showed that in adolescent athletes, tendon strain was significantly higher in symptomatic versus asymptomatic individuals with the risk ratio for developing symptoms being 2.3-fold higher in athletes with a tendon strain ≥ 9% (high strain). However, no differences in tendon forces or stiffness were found between symptomatic versus asymptomatic individuals. Recent evidence from this group also showed that there were no sex differences in adolescent male and female individuals between imbalances in muscle strength and PT stiffness (higher strength with a less stiff tendon results in a high tendon strain), with young female individuals having lower strength and stiffness, and both sexes operating at similar tendon strains [30].

### Interaction Between Morphology and Mechanical Properties

Tendon properties may be confounded by muscle and limb morphology when not normalised to their dimensions. Normalisation refers to the process of attempting to remove bias or errors to establish a true biological signal. An example of normalisation with respect to sex differences in tendon properties would be calculating the PT length relative to the femur length or tendon CSA relative to body mass between sexes. This would establish if the PT length or CSA is truly different between sexes or simply an artifact of scaling between differing sizes of individuals, which often exists at baseline between sexes [22]. Therefore, for example, if tendon properties are calculated using the dimensions such as tendon length or CSA, not normalising them first may affect the interpretation of the observed differences between sexes. An example of this is also provided in the previous section with the work by O’Brien et al. [24]. The mechanical properties of different Achilles morphologies such as a low inserting soleus, additional tendon tissue from an invaginated plantaris (and how this might contribute to load transfer) [31] and AT length in relation to leg length will likely affect mechanical properties. It is not known if any of these morphologies are impacted to a greater degree because of sex.

## What are the Physiological and Biomechanical Differences in Tendon Responses and Adaptation Between Men and Women?

### Acute Responses of Tendons to Mechanical Loading and the Effect of Sex

Tendon metabolism is substantial and adaptable to exercise [32]. Type 1 collagen synthesis in the AT (measured peritendinously) is significantly elevated after long-distance running, whereas markers of collagen breakdown remain unchanged [33]. The PT responds similarly, where for instance the collagen fractional synthetic rate in male individuals following a single leg kicking exercise was significantly elevated 6 h post-exercise, peaked at 24 h and remained elevated compared with baseline at 72 h post-exercise [34].

There are important differences in the resting and acute response to mechanical loading in the key structural and regulatory proteins of the tendon and extracellular matrix of male and female individuals. Patellar tendon tendon dry mass has been found to be lower in female individuals, which also leads to reduced collagen per tendon wet weight in female individuals [35]. The PT responses of male and female individuals differ, female individuals had a lower tendon collagen fractional synthetic rate at rest than male individuals [2]. Interestingly, the collagen fractional synthetic rate was not significantly elevated above baseline values 72 h post-exercise in female individuals yet remained elevated in male individuals [34]. Type I collagen is the key to a tendon’s tensile strength, accounting for 70–80% of its dry mass, with its gene expression and protein production upregulated following physical activity [36]. A change in collagen production is one of the key adaptive mechanisms to overload in tendons [37]. Although collagen type I messenger RNA expression was the same between sexes at rest, collagen type III messenger RNA was significantly higher in female individuals [3]. Type III collagen expression and protein content have been associated with tendon injury and are potentially an indicator of tendon injury [38–40]. Compared with type I collagen, type III collagen forms smaller and less organised fibrils, potentially with an increased susceptibility to rupture [3, 40]. Additionally, there was a trend for *3* metalloproteinase-3 to be lower at rest in female individuals compared with male individuals [3]. Metalloproteinase-3 is up-regulated in response to physical activity and is thought to be a key regulator of healthy tendon extracellular matrix [41]. Injured and ruptured tendons display down-regulation of *3* metalloproteinase-3 in terms of protein content and gene expression [42–44]. These data reflect that female individuals display either baseline or physiological responses to activity that mirror conditions present in injured tendons, therefore providing a potential physiological link to an increased tendon injury risk in female individuals [3].

There are sex differences in how the mechanical properties of tendons are modulated following an acute bout of physical activity. Passive stretching of the AT resulted in a larger decrease in gastrocnemius tendon stiffness, modulus and hysteresis in female individuals compared with male individuals [11]. Additionally, it has been found that female individuals had increased tendon elongation, strain and lower stiffness compared with male individuals at rest, with no changes in response to a jumping exercise task at 20% body mass [5]. However, following more strenuous plyometric exercise, AT stiffness and modulus were significantly reduced compared with baseline in female individuals but not male individuals, with a concomitant increase in AT elongation in female individuals but no change in male individuals [5]. Furthermore, it has been shown that although AT force, stress, strain, stiffness and modulus were all significantly greater in male individuals pre-exercise and post- exercise, there was no sex-specific response (i.e. sex × time interaction) of tendon mechanical properties to 100 consecutive calf raises [12]. These data clearly demonstrate divergence in both internal physiological tendon responses and acute task-specific tendon mechanical behaviour between sexes.

### Adaptability of Tendons to Exercise Training and Habitual Activity

If acute tendon responses to mechanical loading vary between male and female individuals, does the response to habitual activity or exercise also differ? Patellar tendon CSA and stiffness, as well as AT distal CSA, were all significantly greater in male runners compared with both female runners and non-runners [4]. Additionally, the structural and mechanical properties of female runner’s tendons were not significantly different to age-matched non-running controls [4]. In contrast, some studies have shown that male runners had significantly greater AT CSA than male non-runners [45, 46]. No differences are seen in the mean resistance training-induced change in PT stiffness across all force levels between male and female individuals [10]. However, in both younger [10] and older populations [20], female individuals had a significantly greater change in stiffness at the lower portion of the tendon force–elongation curve, whereas male individuals had a significantly greater change in stiffness at the higher portion of the curve. Conversely, mean changes in PT stiffness and PT stiffness at a normalised force level were significantly greater in older male individuals compared with older female individuals following longitudinal loading [20, 22]. Additionally, it has been found that middle-aged male individuals with mid-portion Achilles tendinopathy had a greater reduction in pain and improved outcome measures (Victorian Institute of Sport Assessment - Achilles [VISA-A] scale and Foot and Ankle Outcome Scores) compared with female individuals following 12 weeks of eccentric training, despite female individuals displaying superior adaptations in tendon micro-circulatory variables [47]. This evidence suggests the ability of tendons to adapt to mechanical habitual loading over time appears to be different in character or nature in female individuals compared with male individuals.

## Influences of Measurement, Interpretation of Findings, Puberty and Epidemiology in Identifying Sex-Based Differences in Tendon Health

### Clinical Measures of Response to Loading

Several studies have examined the short-term and long-term effects of energy storage and release loads (highest tendon load) on normal AT in men using algorithmic-enhanced ultrasound [48]. One game of football induced changes in the tendon collagen structure at 2 days post-game that returned to normal at day 4 [49]. Whether this change is normal, adaptive or potentially a response to damage is not known and often not considered in research. Over 3 months of standard pre-season training, 90% of male tendons included in this study improved their structure; however, in a small group, collagen structure deteriorated [50]. The reason for the different response despite the same training load is not known but may reflect base mechanical properties of muscle capacity. There have been no comparative studies in female individuals.

### Limitations of Measures of Tendon Response to Loading and Impact on Evaluating Sex-Based Differences

Normal tendon is maintained through both structural and mechanical adaptations to load. However, measurements are often taken in surrounding tissues (peritendon, aponeuroses) and may not reflect what is occurring in the tendon itself. This has implications when considered in conjunction with research, which has shown that the lengthening that occurs during energy storage and release loads happens in the interfasicular matrix (intra-tendinous connective tissue or endotendon) with a slide and rotation movement [51]. The tendon fascicles do not show a change in length, suggesting that the main load occurs in the intra-tendinous connective tissue and not in the tendon fascicles [51]. Tendon connective tissue is quite different to true tendon tissue in its collagen and proteoglycan content and matrix structure, and its response to load is different. In vitro cell response in culture will in many cases include both tenocytes and connective tissue cells and in vivo responses measured in the peritendon will mainly measure the connective tissue response [52, 53].

Different tendon loads may provoke a different response. This discussion only considers tensile loads and in particular energy storage and release loads. Although these loads are associated with normal tendon adaptation, there are two factors that complicate the issue. Compressive loads are associated with tendon pathology [54, 55], which is also indistinguishable from pathology induced by tensile loads [56]. Biomechanical interpretation of the role of tensile loading’s contribution to the development of tendon pathology is often unclear, contradictory and lacks a clear mechanistic explanation. As mentioned previously outlined in the review, it has been suggested that higher tendon lengthening in women is a protective mechanism against the development of Achilles tendinopathy, citing the lower incidence of tendinopathy found in female individuals [5]. Conversely, it has been suggested that a higher operating strain during activity (i.e. routinely operating closer to the ultimate strain) may increase the potential risk of developing tendinopathy in female individuals [26]. However, neither of these assertions nor other interpretations have been evidenced in vivo in adult humans and are lacking in substantive data.

### Epidemiological Differences

Research across multiple sporting codes has found that PT tendinopathy is twice as prevalent in male individuals compared with female individuals [6, 7]. It is proposed that this difference is due to a reduced force-generating capacity of the knee extensors in female individuals, thereby reducing the amount of force transmitted through the PT [57]. Investigations of jumping and landing kinematics of male and female volleyball players found that when participants were matched for jump height, they generated similar PT loads irrespective of sex [58]. However, even male and female individuals of similar athleticism and loading exposure also demonstrate differences in the PT load. For example, female individuals jump significantly less frequently than men in a similar training environment [57]. It is uncertain if this is because female individuals have less power and therefore strategise to jump less, or if female individuals have less knee extensor power because they jump less often.

In the AT, there appears to be conflicting evidence on the sex-specific prevalence of tendinopathy. For example, in a recent review, Wang and colleagues [8] found no differences between male and female individuals in AT tendinopathy prevalence; however, previous studies found tendinopathy and the incidence of tendon rupture to be higher in younger male individuals [59] and in male individuals versus female individuals across a series of athletic and non-athletic cohorts [60–63]. However, the development of AT tendinopathy may be dependent on other independent factors such as age and physical activity level.

## Perspective

More research across tendon physiology, biomechanics and sports medicine domains is needed (cross-sectional differences, acute changes in physiological responses/mechano-behaviour, chronic adaptations, healthy and diseased states). The apparent differences currently observed between sexes are not without limitations and require a stronger mechanistic link to diverging outcomes of tendon health between sexes. Within the continuing research, a consensus agreement of suitable methodological approaches should be considered toward existing measures, or the inclusion of further measures to account for the differences outlined between male and female individuals (e.g. normalisation to body dimensions, tendon biopsies in appropriate areas of tendons). Additionally, attempts to standardise the in vivo measurement of tendon mechanical properties and morphology should also be made across studies [64]. For example, a large variability exists in researchers’ approaches to ultrasound/dynamometry-based testing of tendon mechanical properties which makes direct comparisons between studies very difficult.

## Conclusions

This Current Opinion article has highlighted that differences exist between male and female individuals in tendon mechanical properties and morphology, responses to loading and adaptation to training/physical activity, which may influence tendon health. However, several confounding or unaccounted for factors in research methodology and design mean that any attempt to link sex differences to divergent injury risks is unwarranted, or at best premature. Therefore, more research is needed to address these current limitations.
